# Colon perforation in multiple myeloma patients – A complication of high‐dose steroid treatment

**DOI:** 10.1002/cam4.3507

**Published:** 2020-10-06

**Authors:** Iuliana Vaxman, Abdullah S. Al Saleh, Shaji Kumar, Mishra Nitin, Angela Dispenzieri, Francis Buadi, David Dingli, Martha Lacy, Eli Muchtar, Miriam Hobbs, Amie Fonder, Lisa Hwa, Alissa Visram, Prashant Kapoor, Mustaqeem Siddiqui, John Lust, Robert Kyle, Vincent Rajkumar, Suzanne Hayman, Nelson Leung, Wilson Gonsalves, Taxiarchis Kourelis, Rahma Warsame, Morie A. Gertz

**Affiliations:** ^1^ Division of Hematology Mayo Clinic Rochester MN USA; ^2^ Institute of Hematology Davidoff Cancer Center Rabin Medical Center Petah‐Tikva Israel; ^3^ Sackler Faculty of Medicine Tel‐Aviv University Tel‐Aviv Israel; ^4^ King Saud bin Abdulaziz University for Health Sciences Riyadh Saudi Arabia; ^5^ Division of Colon and Rectal Surgery Mayo Clinic Scottsda AZ USA

**Keywords:** colostomy, diverticulitis, intestinal, multiple myeloma, perforation

## Abstract

Gastrointestinal complications of multiple myeloma (MM) treatment are common and include nausea, constipation, and diarrhea. However, acute gastrointestinal events like perforations are rare. We aimed to describe the characteristics and outcomes of patients with MM that had colonic perforations during their treatment. This is a retrospective study that included patients from all three Mayo Clinic sites who had MM and developed a colonic perforation. All patients were diagnosed with colonic perforations based on CT scans and were surgically treated. Patients diagnosed with AL amyloidosis, a perforated colon complicating neutropenic colitis during ASCT and those with perforation due to colonic cancer were excluded. A high dose of dexamethasone was defined as ≥40 mg dexamethasone once a week. Thirty patients met inclusion criteria. All patients received steroids at doses ≥10 mg once weekly prior to the perforation, while four (11%) were on high‐dose dexamethasone without chemotherapy. Fourteen patients were given high doses of dexamethasone. Twenty‐five patients required ostomies with all surviving surgery. Twenty‐four perforations (80%) were associated with diverticulitis. Treatment with steroids was resumed in 23 patients with no further gastrointestinal complications. The median OS was 20 months following perforation (IQR 8–59). Within the same timeframe 5854 patients were treated at Mayo Clinic for MM, making the risk of bowel perforation 0.5%. Intestinal perforations in MM are rare and, in our series, always occurred with dexamethasone ≥10 mg per week. Urgent surgery is lifesaving and resumption of anti‐myeloma treatment appears to be safe.

## Graphical abstract

Colon perforations are rare in MM and here we report characteristics and clinical outcomes of 30 patients that were treated at Mayo Clinic. We believe that steroids are the precipitating factor.

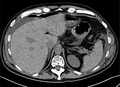

## 1 INTRODUCTION

Multiple myeloma (MM) is a systemic disease characterized by renal failure, lytic bone lesions, anemia, and hypercalcemia.^1^ It is commonly treated with antimyeloma agents including alkylating agents, proteasome inhibitors, immunomodulatory agents, and monoclonal antibodies.^2^, ^3^, ^4^ All therapeutic agents used to treat MM can cause gastrointestinal (GI) side effects, including anorexia, nausea, constipation, and diarrhea.^5^, ^6^ However, acute GI events such as hemorrhage^7^, ^8^ or perforation^9^, ^10^ are rare and have been reported only in case reports and small case series.

High‐dose corticosteroids are the backbone of treatment for MM in both the frontline and the relapsed/refractory setting.^11^ Ulceration and perforation of the duodenum and stomach are well recognized complications of corticosteroid therapy. However, awareness of colon perforations related to steroid treatment is low. Moreover, the clinical signs and symptoms of bowel perforation may be obscured by the anti‐inflammatory effects of steroids^12^ thereby delaying the diagnosis and surgical intervention. The risk of poor wound healing in patients receiving steroids^13^ is substantial and poses further challenges to the treating physician. The reported death rates for GI perforations in patients receiving steroids were above 80%.^14^

This study reports the characteristics and outcomes of MM patients that were treated for colonic perforations at Mayo Clinic.

## 2 METHODS

We retrospectively identified patients treated at all three Mayo Clinic sites who had MM and developed a gastrointestinal perforation. The study was approved by the Mayo Clinic Institutional Review Board (IRB). The diagnosis and staging of MM were according to consensus criteria.^15^ All patients were diagnosed with GI perforations based on computerized tomography (CT) scans and treated surgically. Patients with AL amyloidosis, a perforated colon complicating neutropenic colitis during autologous stem cell transplantation (ASCT), or those with a perforation due to colonic cancer were excluded because those conditions can independently cause perforation. High‐dose steroids were defined as greater than 40 mg dexamethasone weekly. Overall survival was defined as time from MM diagnosis to death of any cause. Statistical analysis was carried out using JMP 14 (SAS Institute) statistical software.

## 3 RESULTS

We identified 76 patients with MM who developed gastrointestinal perforations between January 1997 and February 2020. Forty‐six were excluded due to the following reasons: perforations due to colonic carcinoma,^7^ concomitant AL amyloidosis,^16^ graft versus host disease (GVHD) involving the GI tract,^8^ bowel perforations in the thirty days posttransplant period with neutropenic colitis diagnosed per CT,^9^ clostridium colitis,^2^ and invasive cytomegalovirus (CMV) of the colon.^2^ Overall, 30 patients with MM with GI perforations were included in the analysis and are presented in Table 1. The median age at GI perforation was 66 years (IQR 60–71). Twenty‐two (70%) were male. The median time from diagnosis of MM to perforation was 4 months (IQR 2–28). Eleven patients were treated with oxycodone prior to the perforation and 6 patients had peripheral neuropathy prior to perforation. Fourteen patients (47%) were treated with bortezomib at the time of perforation. The regimens varied and are reported in Table 1. Four patients underwent ASCT, all more than a year prior to perforation. Of note, four patients were on high‐dose dexamethasone without chemotherapy at the time of perforation. Twenty patients perforated during first‐line therapy, one during second line, five during third line, two during fourth line, and two during fifth line of therapy. The median duration from the initiation of the current line of treatment to perforation was 2 months (IQR 1–4).

**TABLE 1:** MM patient with GI perforations reported in the article

Pt	Age/gender	Number of treatment lines prior to perforation	Months from diagnosis to perforation	Dexamethasone dose	Regimen while perforation
1	68/F	3	72	20 mg weekly	Anakinra+bortezomib+dexamethasone
2	70/M	1	1	40 mg daily for 14 days	IV melphalan+dexamethasone
3	59/M	5	86	40 mg 3 days, every week	Dexamethasone only
4	71/F	4	124	40 mg weekly	Bortezomib+dexamethasone
5	63/M	1	3	20 mg weekly	Lenalidomide+dexamethasone
6	57/M	1	11	40 mg 4 days on and 4 days off	C‐VAD 6 cycles+cyclophosphamide+dexamethasone for 7 cycles
7	79/M	1	1	40 mg 3 days on 3 days off	Bortezomib+cyclophosphamide+dexamethasone
8	44/M	1	2	40 mg weekly	Bortezomib+lenalidomide+dexamethasone
9	46/F	4	40	40 mg 4 days once every 3 weeks	VDTPACE
10	60/M	1	1	40 mg weekly	Bortezomib+cyclophosphamide+dexamethasone
11	76/F	1	18	40 mg weekly	Bortezomib+dexamethasone
12	70/F	1	24	10 mg weekly	Bortezomib+cyclophosphamide+dexamethasone
13	66/M	3	14	20 mg weekly	Daratumumab+pomalidomide+dexamethasone
14	57/M	3	71	20 mg weekly	Thalidomide+dexamethasone
15	57/M	5	42	Prednisone 100 mg 5 days every month	HD Cytoxan+prednisone
16	49/M	3	83	40 mg 4 days on and 4 days off	VAD
17	66/F	3	4	40 mg 4 days on and 4 days off	VDTPACE
18	66/M	1	1	40 mg 4 days on and 4 days off	Lenalidomide+dexamethasone dexamethasone
19	66/M	2	4	40 mg 4 days on and 4 days off	Bortezomib+lenalidomide+dexamethasone+cytoxan
20	61/M	1	6	40 mg weekly	Bortezomib+lenalidomide+dexamethasone
21	70/M	1	2	40 mg 4 days on and 4 days off	Dexamethasone only
22	78/M	1	2	40 mg weekly	Bortezomib+cyclophosphamide+dexamethasone
23	61/M	1	14	40 mg 4 days on and 4 days off	Thalidomide+dexamethasone
24	54/F	1	3	40 mg 4 days on and 4 days off	Dexamethasone only
25	54/F	1	2	40 mg 4 days on and 4 days off	VAD
26	65/M	1	3	40 mg 4 days on and 4 days off	Thalidomide+dexamethasone
27	40/M	1	0	40 mg 4 days on and 4 days off	Dexamethasone only
28	83/M	1	7	40 mg weekly	Lenalidomide+dexamethasone
29	66/M	3	4	40 mg weekly	Bortezomib+lenalidomide+dexamethasone+cyclophosphamide
30	68/M	1	1	40 mg weekly	Bortezomib+lenalidomide+dexamethasone

Abbreviations: C‐VAD, cyclophosphamide, vincristine, doxorubicin, dexamethasone; F, female; M, male; VDTPACE, bortezomib, dexamethasone, thalidomide, cisplatin, doxorubicin, cyclophosphamide, etoposide.

All 30 patients received dexamethasone as part of their treatment with doses ranging from 10 mg once weekly to 40 mg day 1–4, 9–12, 17–20 q 28 days. Fourteen patients received very high doses of steroids (dexamethasone 40 mg day 1–4, 9–12, 17–20 q 28 days) and 23 patents (77%) received high‐dose corticosteroids (≥ dexamethasone 40 mg weekly). One patient received only prednisone and not dexamethasone. The median cumulative dose of dexamethasone given prior to perforation was 680 mg (IQR 240–1760).

Four patients were on dialysis prior to perforation. Coexisting autoimmune conditions (ulcerative colitis, scleroderma) which might have contributed to the development of colonic perforations were not present in our cohort.

Thirteen patients presented with an acute onset (24–48 h) of symptoms (e.g., abdominal pain, nausea). One patient presented with abdominal pain and septic shock, one patient presented with fever and cough without abdominal pain and one patient presented with septic shock without abdominal symptoms. One patient presented with 1 week of abdominal pain and diarrhea. One patient was asymptomatic and free abdominal fluid was seen on CT done for other reasons. Data about the presentation of 12 patients were missing.

**FIGURE 1:**
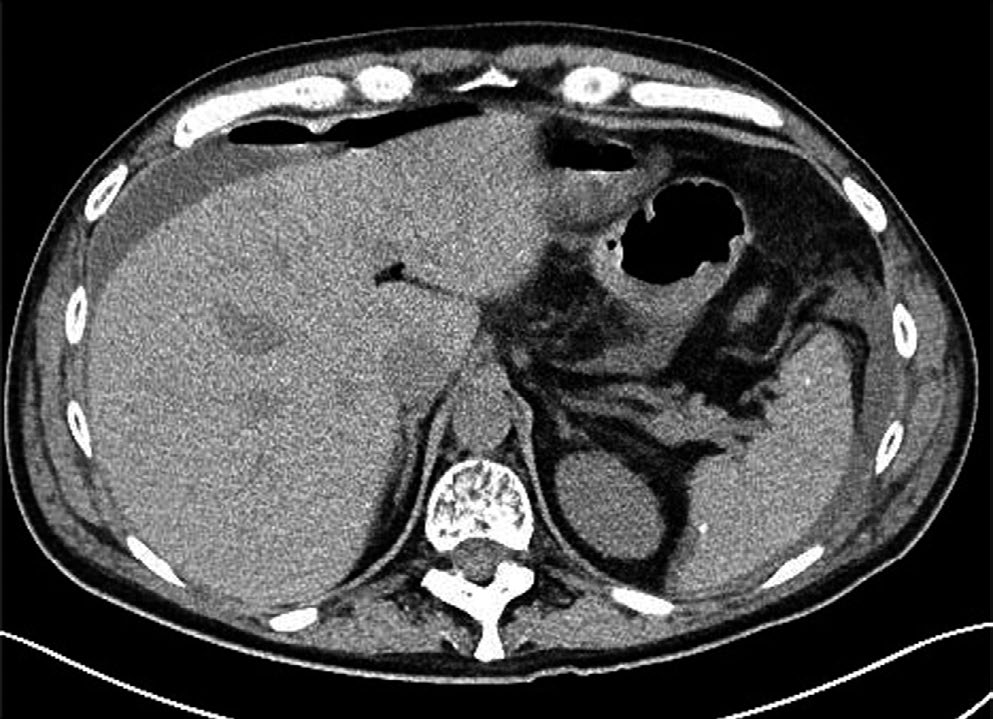
Bowel perforation in a CT scan in one of the patients Courtesy of Benjamin M. Howe

Perforation involved the colon in all 30 patients. All patients underwent surgical intervention for the perforated colon. Three patients had ileostomy and 21 had a colostomy (operative notes of one additional patient were missing). Five patients did not need stomas after bowel resection. The pathological diagnosis were perforated diverticulitis in 24 patients, perforated transverse colon in one patient and perforation was located to the transverse colon in one patient. One patient had ischemic colitis and the entire colon was resected. Pathology reports were unavailable in three patients with a known colon perforation. None of the patients had evidence of bowel infiltration with plasma cells. Congo red stain was not done in the pathological specimens. Urgent surgery was successful and the postoperative recovery was uneventful in 26 patients. One patient developed acute renal failure needing dialysis and two patients had sepsis causing death less than a week post‐surgery. One patient developed ischemic colitis and died 74 days after surgery. Of 15 patients with available data about length of hospitalization, median length of hospitalization was 8 days (IQR 6–12). One patient had small bowel obstruction due to adhesions after discharge and one developed a colonic fistula. One patient had a recurrent perforation of sigmoid colon a year after the first perforation and needed an additional stoma. This patient did not resume treatment for MM between the two episodes. Seven patients underwent colostomy reversal at a median time of 4 months post perforation (IQR 2–12). Figure 1 shows a typical bowel perforation demonstrated in a CT scan (patient number 8).

Twenty‐six patients resumed their previous MM treatment after perforation, 23 (77%) of them continued dexamethasone treatment. No bowel complications were seen after treatment resumed.

Nine patients are currently alive. The median OS from diagnosis and from perforation were 72 months (IQR 39–95) and 20 months (IQR 8–59), respectively. Twenty‐one patients died: six due to progressive disease, three due to infections unrelated to the colon (one necrotizing fasciitis, two sepsis), one due to congestive heart failure, and one due to cerebral hemorrhage. Three patients died of infectious complications of colon surgery: two had sepsis in the first week after perforation and the other had colonic ischemia that led to sepsis less than 100 days after the surgery (operative mortality 10%). The cause of death of the remaining seven patients was unknown. Table 2 summarizes the outcomes of this cohort.

**TABLE 2:** Outcomes of the cohort

Outcome	(N = 30)
Median time from MM diganosis to perforation	4 months (IQR 2–28)
Median time from initiation of current line of treatment to perforation	2 months (IQR 1–4)
Requiring ostomies	25 patients
Patholigical diagnosis of diverticulitis	24 patients
Median overall survivial following perforation	20 months
Stoma Closure	7 patients
Hospital admission length	8 days (IQR)
Death as a result of postoperative complications	3 patients (IQR 6–12)
Resuming MM treatment	26 patients
Colostomy reversal	7 patients

In the same timeframe (1997–2000) 5854 patients were treated at Mayo Clinic for MM, making the risk of bowel perforation 0.5%. The median OS of the cohort that did not undergo bowel perforation was 98.6 months. This was not different than the 72 months OS that we found in the cohort of patients that underwent perforation (*p* = 0.12).

## 4 DISCUSSION

This is a report of 30 patients with MM and colon perforation while receiving treatment for MM. All patients were treated with corticosteroids at doses higher than dexamethasone 10 mg per week prior to perforation and 87% had good recovery. Twenty‐six patients resumed treatment without further GI complications, 23 also resumed dexamethasone.

In a literature review, we found few reports concerning bowel perforations in MM patients. In a case series of 84 patients treated with thalidomide, two patients had MM and presented with an acute abdomen requiring surgical intervention for bowel perforation. Both were treated with dexamethasone and none of these patients were treated with bortezomib or vincristine.^17^ Perforations were ascribed to thalidomide treatment (autonomic neuropathy causing slower peristalsis or impaired wound healing). A second case series reported six MM patients treated with thalidomide that developed diverticular disease associated bowel perforation. All of them received dexamethasone (20–40 mg once weekly) and one also received bortezomib.^9^ One case report described a MM patient on dexamethasone and morphine with bowel perforation due to diverticulitis.^18^ Another case report of a 61‐year‐old patient treated with bortezomib, lenalidomide, and dexamethasone (VRd) that developed peritonitis secondary to a cecal perforation and perforation was ascribed to lenalidomide treatment.^16^ We could not find reports of perforation in MM patients treated with melphalan and prednisone, used routinely to treat myeloma before the use of novel agents.

In AL amyloidosis, intestinal involvement is present in 15% of the patients^19^ and gastrointestinal perforations have previously been reported.^20^ A case series reported seven patients with bowel perforations, all of them were treated with dexamethasone as part of their treatment regimen prior to perforation. One had diverticulitis and all had amyloid deposition in their pathological specimen.^20^ In this cohort, treatment was resumed in four patients without further GI complications.

In 1950, the first two cases of colonic perforations in patients treated with adrenocorticotrophic hormone (ACTH) were published.^21^ Subsequently more cases linking corticosteroids and bowel perforation were reported.^22^, ^23^, ^24^ Moreover, cases that reported the association of colonic diverticulitis and corticosteroids have been published.^25^, ^26^, ^27^, ^28^, ^29^

A masking effect of steroids can cause patients to present with more advanced disease. Failure to correctly identify perforations has been previously proven to be a cofactor in the elevated mortality rates that were reported in patients that perforated while receiving steroids.^14^, ^29^ A retrospective study that evaluated complications of diverticular disease demonstrated that patients receiving steroids are at higher risk of severe septic complications of diverticular disease (fistula, extra colonic abscess, purulent peritonitis, fecal peritonitis, and sepsis).^30^ Another retrospective study that reported GI perforations in 79 patients treated with steroids, showed that patients receiving higher doses of steroids had a mortality rate of 85%. The clinical expression of peritonitis was attenuated in the high‐dose steroid subgroup, causing significant delays in recognition and treatment.^14^ Another retrospective trial reported 13 patients (one with MM) with colonic perforations while treated with steroids. One patient was asymptomatic, and three patients had only minimal abdominal tenderness. Eight patients had perforated sigmoid colon and five perforated in unusual locations (cecum, hepatic flexure, and multiple perforations along the transverse and descending colon).^26^ In our cohort, two patients presented without abdominal symptoms and one patient was asymptomatic and free abdominal fluid was seen in CT scans done for other reasons. We showed that perforation do not appear to affect OS which was similar to a cohort treated at Mayo Clinic at the same timeframe.

The association between bowel perforations and steroids in other fields of medicine in which high‐dose corticosteroids are used, such as neurologic patients, has been reported. A retrospective study of 107 patients with spinal cord compression that received dexamethasone 16 mg/day reported three patients with bowel perforations. Rectosigmoid perforations and associated constipation were more frequent in patients that received steroids than in patients with bowel perforations that did not receive steroids. Bowel perforations were more frequent than GI bleeding, a well‐recognized side effect of steroid treatment.^31^ A case series of three patients with myasthenia gravis treated with high doses of steroids also supported the association between steroids and perforations of colonic diverticula.^28^ In all three patients, perforations developed within the first 16 days of initiation of steroid treatment. In our cohort, 24 out of 30 patients (80%) had bowel perforation due to diverticulitis.

Gastrointestinal perforations have also been reported in three lymphoma patients that were treated with chemotherapy and steroids.^32^ Corticosteroids are an integral part of MM treatment regimens, and with the paradigm of continuous lifelong MM treatment being the standard of care, MM patients receive higher doses of corticosteroids than any other hematological malignancy. Therefore, the risk of colon perforations in MM may be higher than other hematologic malignancies.

The link between corticosteroid treatment and diverticulitis causing colon perforations in MM was previously reported. The first is a retrospective report of outcomes of 30 cancer patients (one MM patient) with spontaneous intestinal perforation. About 76% received steroids alone or with chemotherapy.^33^ The second was a report of bowel perforation in two patients, one had MM treated with steroids and the other received long‐term low‐dose steroid treatment for asthma.^34^ In MM, high‐dose dexamethasone is a fundamental part of all treatment regimens. For MM patients with perforated colon, some clinicians postulate that bortezomib is the culprit,^20^ but we believe, although cannot prove, that although bortezomib and immunomodulatory agents can cause neuropathies^35^, ^36^ and influence bowel motility,^6^ steroids are the cause of the perforation. Fourteen patients (46%) received very high doses of steroids (dexamethasone 40 mg day 1–4, 9–12, 17–20 q 28 days) and 23 patents (77%) received high‐dose steroids (defined as a dose higher than 40 mg/week) so we are unable to show that the perforations are related to the dose of dexamethasone. We cannot unequivocally exclude other reasons for the perforations but the association between colon perforations and steroids is well established in the surgical literature.

Acquired diverticula of the colon are epidemiologically linked to advanced age and lifestyle and diet factors.^37^, ^38^, ^39^ A minority of the patients develop acute inflammatory complications that range from uncomplicated diverticulitis to localized or generalized peritonitis. The pathogenesis of corticosteroid associated bowel perforation is unknown. Steroids impair mucosal renewal, enabling bacteria to penetrate the mucosal barrier. Colonic diverticula are sites of high concentrations of bacteria due to stasis, which would predispose to bacterial translocation through the mucosa and submucosa. The lack of muscularis in the diverticula makes this area more vulnerable to perforations. Steroids also impair the inflammatory response, diminishing host defenses.

It may be that diverticular disease or other local bowel pathologies predispose to perforation when MM patients are treated with high doses of steroids. Early diagnosis and treatment are important to improve the prognosis of bowel perforation. Constipation should be correctly managed with appropriate laxatives, especially in patients that are also treated with opiates. When addressing renewal of treatment in patients treated for MM with combination treatments, one should consider steroids as a plausible cause of bowel perforations and consider dose reductions in patients that have already responded to MM therapy. Perforations of the colon must be added to the list of adverse effects of MM treatments. Because the clinical manifestations may be obscured, abdominal complaints in MM patients treated with steroids should be addressed seriously and promptly.
